# Rapid Deterioration of Glycaemic Control Revealing Interval Metastatic Pancreatic Cancer Despite a Recent Normal CT Scan

**DOI:** 10.7759/cureus.115088

**Published:** 2026-08-24

**Authors:** Fatima Alam, Suham Amin, Ekram Omer, Nafisa Nasser, Muhammad A Alam

**Affiliations:** 1 Acute Medicine, Stockport NHS Foundation Trust, Manchester, GBR; 2 Acute Medicine, Stepping Hill Hospital, Stockport, GBR; 3 General Surgery, Services Hospital Lahore, Lahore, PAK

**Keywords:** glucose, hyperglycaemia, metabolism, metastatic disease, pancreatic cancer, rapid progression

## Abstract

New-onset diabetes in the elderly or acute deterioration of glycaemic control in already known diabetics is sometimes linked with suspicion of pancreatic malignancy. We report the case of an 81-year-old woman with long-standing, type 2 diabetes, well-controlled on gliclazide, who developed worsening glycaemic control over four months. A contrast-enhanced CT scan performed for an unrelated indication four months earlier was reported as normal. She presented with symptomatic hyperglycaemia with acutely deranged capillary blood sugar levels and a markedly elevated glycosylated haemoglobin (HbA1c), without evidence of infection or other precipitating factors. Repeat imaging demonstrated a necrotic pancreatic mass with metastatic disease and concurrent saddle pulmonary embolism. This case demonstrates that pancreatic malignancy may become apparent despite previously normal imaging and illustrates the diagnostic challenge when longstanding diabetes suddenly deteriorates.

## Introduction

Pancreatic cancer is a highly aggressive malignancy with a poor prognosis, often diagnosed at an advanced stage due to its insidious onset [1]. There is a well-established association between pancreatic cancer and abnormalities in glucose metabolism, mostly new-onset diabetes or worsening glycaemic control [2-4]. Usually, pancreatic cancer in diabetics is associated with weight loss despite initial weight gain that can lead to workup for malignancy [5]. In some cases, diabetes may precede the diagnosis of pancreatic cancer and serve as an early clinical marker [4,6]. Usually, imaging or workup for pancreatic cancer is not done in the general population due to low incidence; however, reliance on recent imaging may provide false reassurance. We present a case of rapid deterioration of glycaemic control in a patient with long-term well-controlled known diabetes, leading to the diagnosis of metastatic pancreatic cancer despite a recent normal CT scan.

## Case presentation

An 81-year-old woman was referred by her general practitioner to the emergency department for assessment of poorly controlled diabetes mellitus and review by the diabetes specialist team.

She had a past medical history of aortic bifurcation syndrome, ischaemic heart disease with coronary artery bypass graft, chronic kidney disease stage 3, osteoarthritis, type 2 diabetes mellitus, essential hypertension, right breast cancer and endometrial cancer.

Her regular medication included gliclazide, ivabradine, candesartan, isosorbide mononitrate, atorvastatin, aspirin, lansoprazole and glyceryl trinitrate spray.

She was previously on metformin for her diabetes control, but her community doctor discontinued it due to deteriorating kidney function and commenced on gliclazide 11 months prior to this presentation.

The patient reported a one-week history of feeling unwell, generalised abdominal pain after eating, increasing fatigue, weight loss and increased thirst. There were no symptoms suggestive of infection, including no cough, sputum production, shortness of breath, chest pain, dysuria, vomiting, or diarrhoea.

Initial blood panel (Table 1) showed mildly elevated white cell count and C-reactive protein. Renal function demonstrated mild deterioration as compared to the patient's baseline chronic kidney disease.

**Table 1:** Initial blood panel. ALP, alkaline phosphatase; ALT, alanine aminotransferase; CRP, C-reactive protein; eGFR, estimated glomerular filtration rate; GGT, gamma-glutamyl transferase; Hb, haemoglobin; HbA1c, glycosylated haemoglobin; INR, international normalised ratio; Plt, platelets; WCC, white cell count.

Test	Result	Normal value/range
Hb	136	115 - 165 g/L
WCC	13.7	3.7 - 11.0 x 10*9/L
Plt	177	150 - 450 x 10^9/l
INR	1.0	-
Serum adjusted calcium	2.4	2.20 - 2.60 mmol/L
Serum phosphate	0.89	0.80 - 1.50 mmol/L
CRP	58	0.0 - 10.0 mg/L
Bilirubin	8	1 - 21 umol/L
ALT	25	< 33 U/L
ALP	151	20 - 130 U/L
GGT	35	< 50 u/L
Sodium	129	133 - 146 mmol/L
Potassium	5.2	3.5 - 5.0 mmol/L
Urea	8.2	2.5 - 7.8 mmol/L
Creatinine	132	45 - 100 umol/L
eGFR	58	> 60 ml/min
Blood glucose	25.0	3.3 - 6.1 mmol/L
Initial ketones	1.4	0.0 - 0.9 mmol/L
Venous pH	7.40	7.35 - 7.45
Venous bicarbonate	23.5	22.0 - 29.0 mmol/L
Venous lactate	1.3	0.6 - 2.0 mmol/L
HbA1c	125	0 - 41 mmol/mol
Lipase	66	12 - 53 U/L

These were stable as compared to her last kidney function tests done four months ago (Table 2).

**Table 2:** Kidney functions done four months ago; background of chronic kidney disease stage 3. eGFR, estimated glomerular filtration rate.

Test	Result	Normal value/range
Urea	8.0	2.5 - 7.8 mmol/L
Creatinine	130	45 - 100 umol/L
eGFR	47	> 60 ml/min

Sodium levels showed acute mild hyponatraemia that was deemed secondary to dehydration. Her blood sugars were high, along with mild ketosis evident on venous blood gas. Isolated rise in alkaline phosphatase (ALP) was considered non-specific in nature and deemed secondary to post-menopausal bone turnover. Her glycosylated haemoglobin (HbA1c) levels were deranged (125), compared with being in range (49) four months ago (Table 3).

**Table 3:** HbA1c done fpur months ago for the patient's usual diabetes check-up. HbA1c, glycosylated haemoglobin.

Test	Result	Normal value/range
HbA1c	49	0-41 mmol/mol

She was managed as hyperglycaemia with ketosis on background of poorly controlled type 2 diabetes mellitus. She was initially commenced on short-acting insulin and fluids with regular monitoring of blood sugars and ketones until blood sugars became <15 mmol/L and ketones <0.6 mmol/L (Table 4). Sodium levels were also monitored after intravenous fluid therapy.

**Table 4:** Corrected sodium levels after fluid therapy.

Test	Result	Normal value/range
Serum sodium	133	133-146 mmol/L

Later, she was reviewed by diabetes specialist team who stopped her gliclazide and commenced her on once a day long acting insulin with three times a day short acting insulin regimen. She was kept under observation, and her insulin was optimised until blood sugars were in the normal range.

Review of prior investigations revealed that she had undergone a contrast-enhanced CT scan of the thorax, abdomen, and pelvis four months prior to this hospital attendance as part of the evaluation for iron deficiency anaemia, which showed no significant abnormalities, including any malignancy or pancreatic pathology.

**Figure 1:**
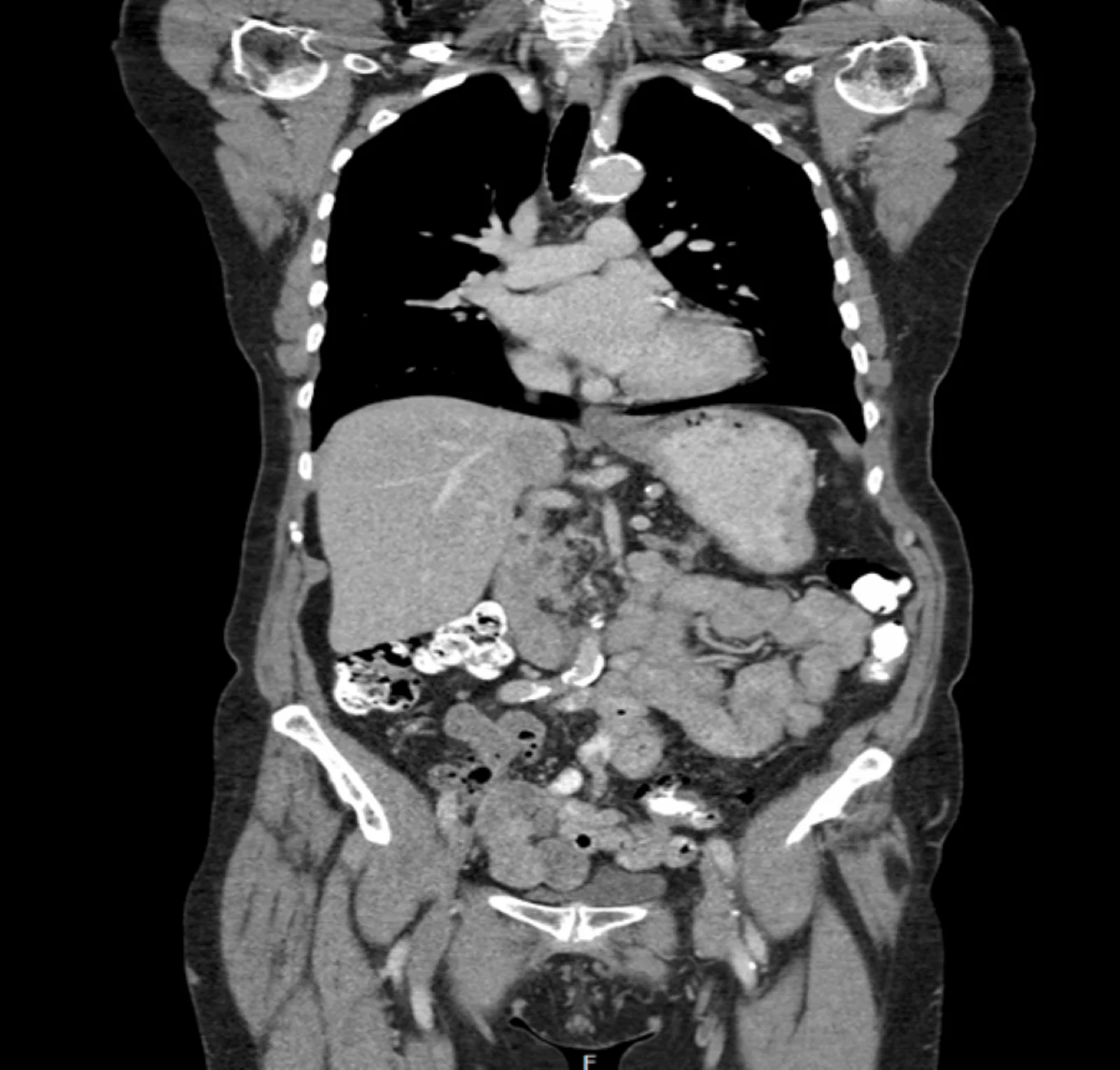
CT of the thorax, abdomen, and pelvis performed four months previously as part of the work-up for potential malignancy due to iron-deficiency anaemia was normal, with no evidence of malignancy or pancreatic pathology.

Figure 1 corresponds to a coronal section of CT thorax, abdomen and pelvis showing no sinister pathology such as cancer or distant metastasis. Lung fields seem to be clear with no evidence of pulmonary embolism.

**Figure 2:**
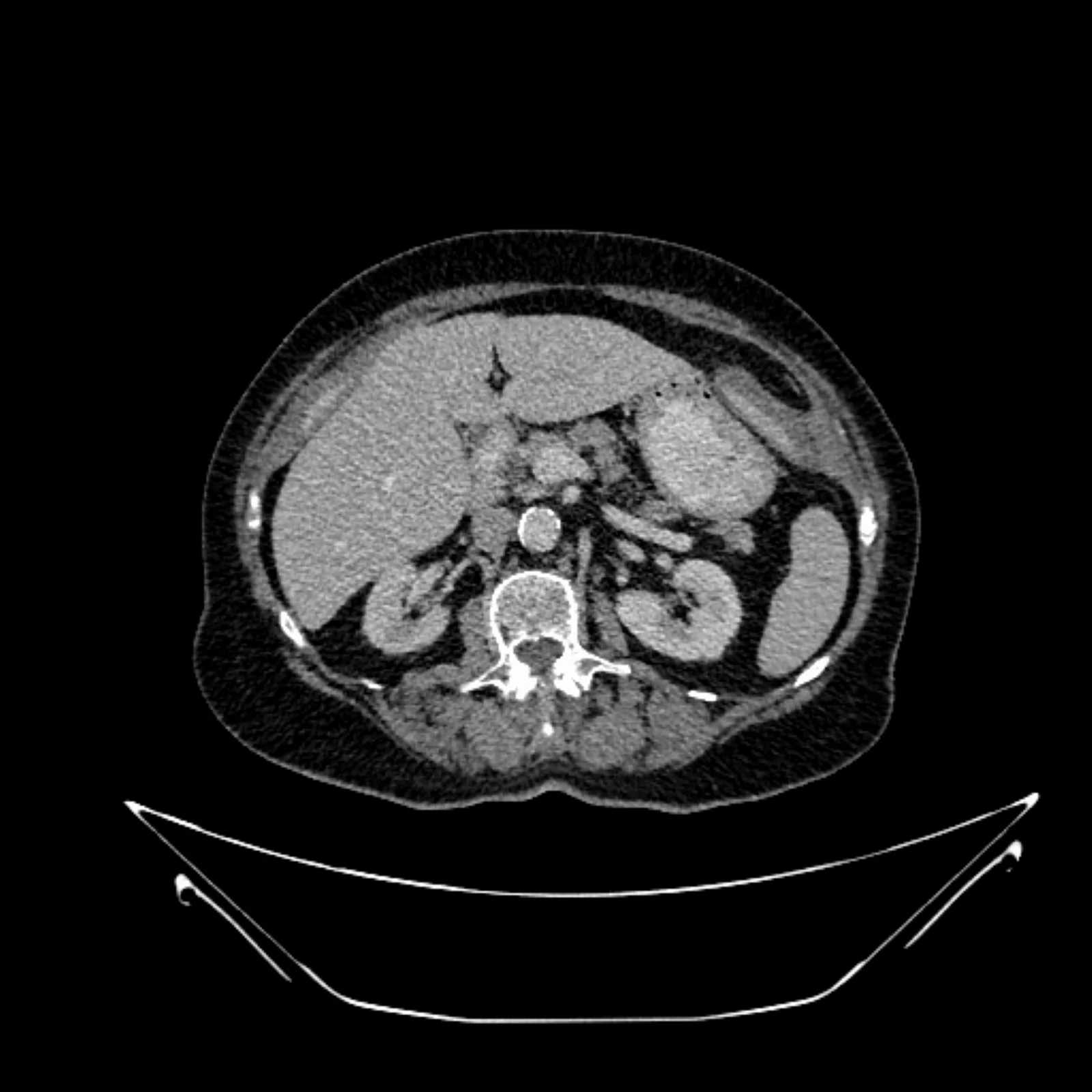
Cross-sectional CT image of the abdomen and pelvis showing a normal liver and pancreas, with no evidence of metastatic disease.

Figure 2 corresponds to a cross-sectional image of the CT abdomen showing the liver and pancreas with no evidence of necrotic mass or metastatic deposits.

**Figure 3:**
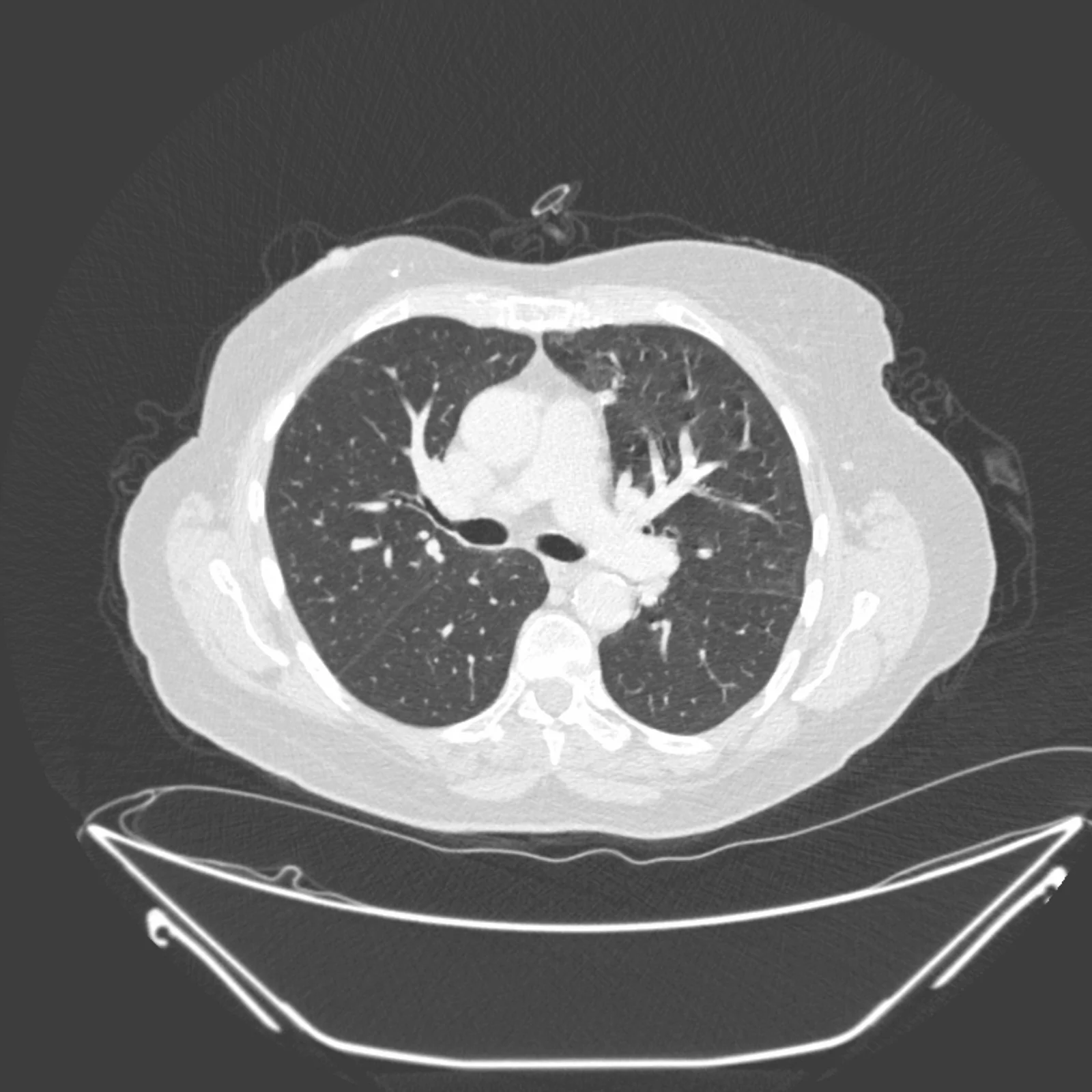
Cross-sectional CT image of the thorax showing no evidence of metastatic disease and good opacification of the pulmonary trunk.

Figure 3 corresponds to a cross-sectional image of the lung fields showing no evidence of metastatic disease or pulmonary embolism.

Given the drastic change in her HbA1c as compared to four months previously (from 49 to 125 mmol/mol), despite full compliance with medication and in the absence of identifiable precipitating factors, pancreatic disease was suspected clinically. This prompted a repeat CT scan of the thorax, abdomen, and pelvis. The formal report demonstrated a saddle pulmonary embolism (PE) without radiological evidence of right heart strain or an associated pulmonary infarct. The PE was considered likely to be provoked by underlying malignancy. The scan also showed features suggestive of pancreatic malignancy, with possible invasion of the proximal portal vein, new common bile duct (CBD) obstruction, and findings suspicious for hepatic metastases, although these were located in an area where focal fatty change is commonly seen. If malignancy were confirmed, the provisional staging was T3, N1, M1. The pulmonary nodules were considered potentially inflammatory changes secondary to multiple PEs, although new metastatic deposits could not be excluded.

**Figure 4:**
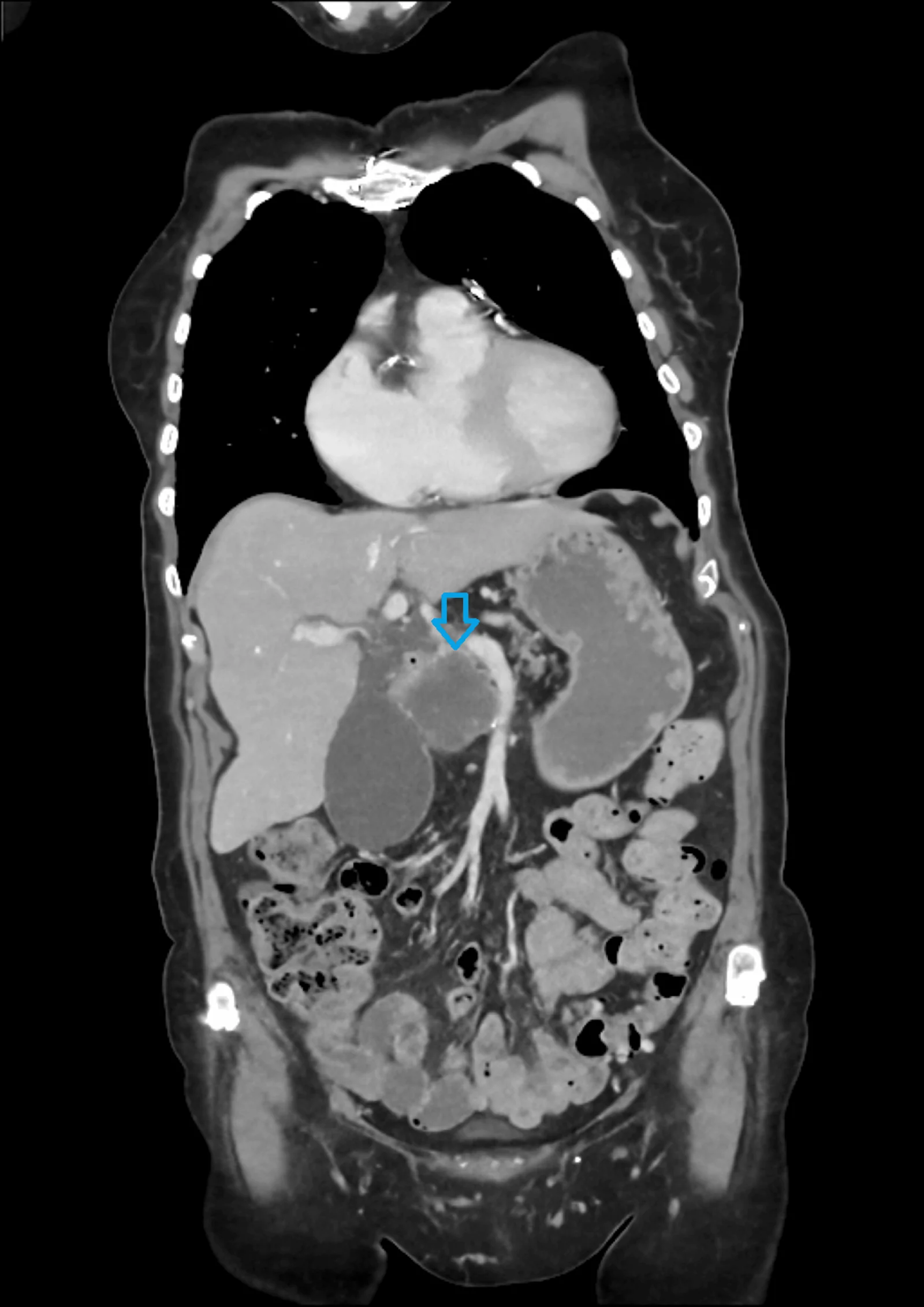
Coronal section of CT thorax, abdomen and pelvis with contrast, with the blue arrow pointing towards large pancreatic mass.

Figure 4 corresponds to a coronal section of CT thorax, abdomen and pelvis showing a large pancreatic mass in the abdominal region.

**Figure 5:**
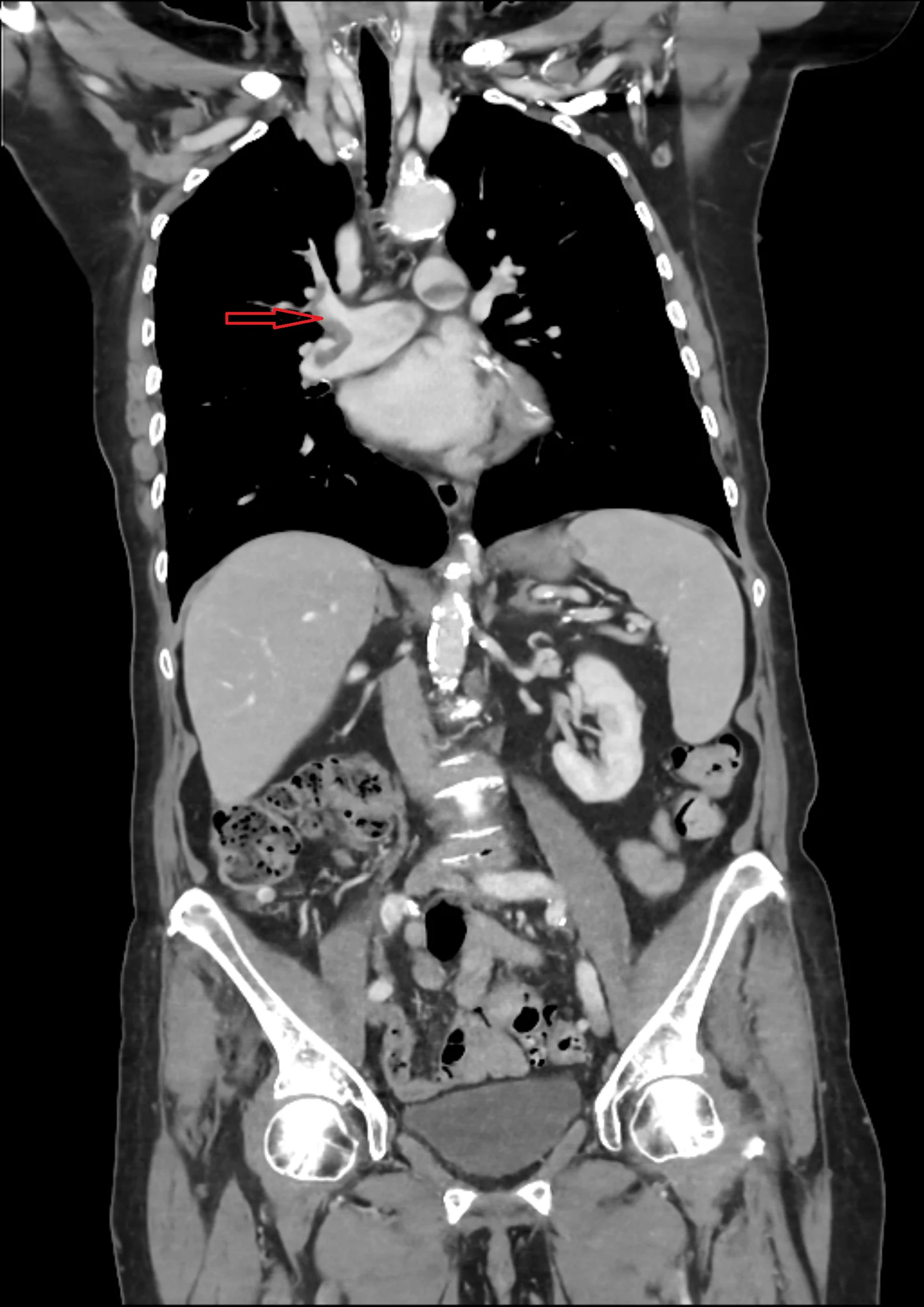
CT thorax, abdomen and pelvis with contrast, with the red arrow pointing towards large pulmonary embolism.

Figure 5 corresponds to a coronal section of CT thorax, abdomen and pelvis, with focus being on the thoracic region showing a large pulmonary embolism.

**Figure 6:**
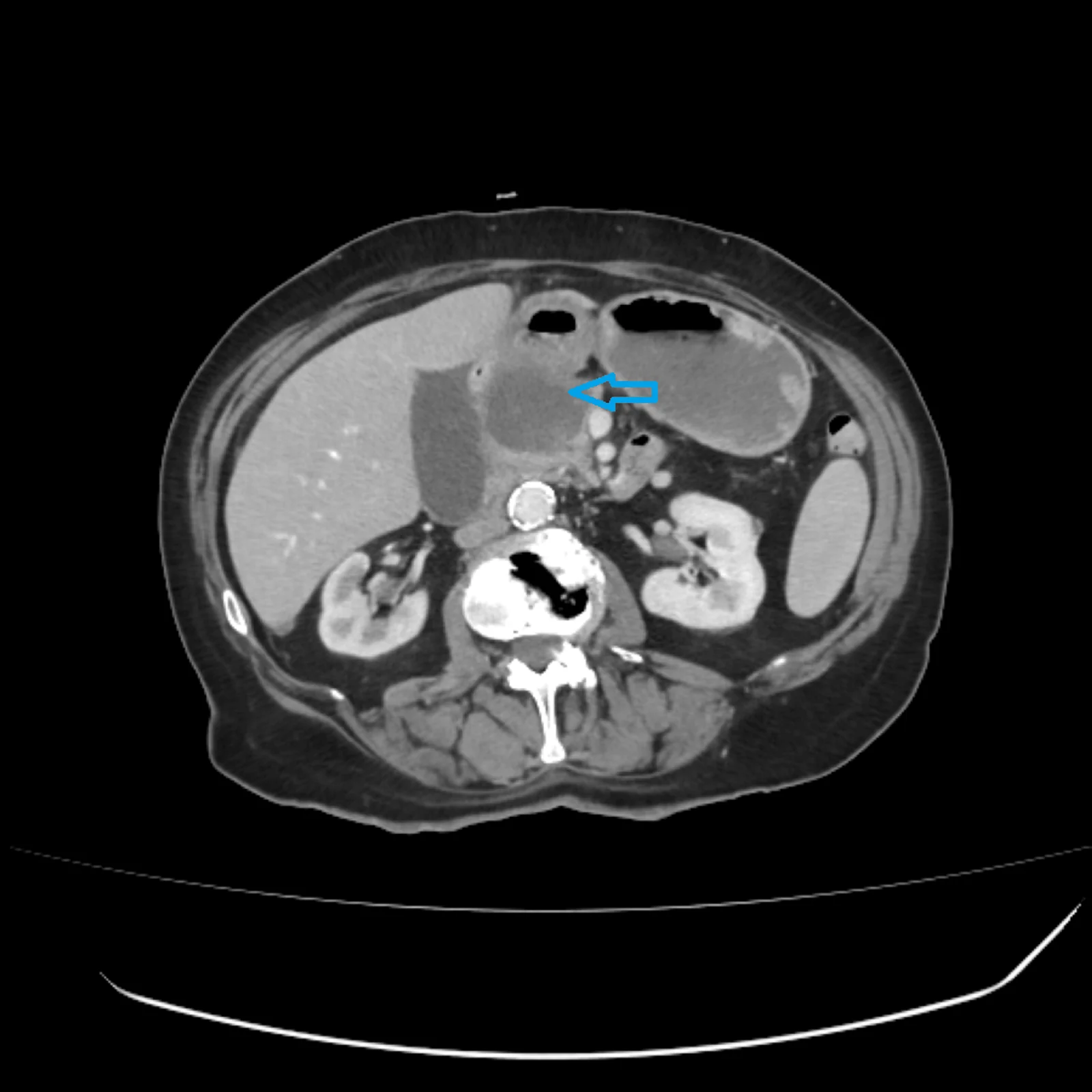
Cross-section of CT abdomen with contrast, with the blue arrow pointing towards large necrotic pancreatic mass.

Figure 6 corresponds to a cross-sectional image of CT abdomen with contrast.

**Figure 7:**
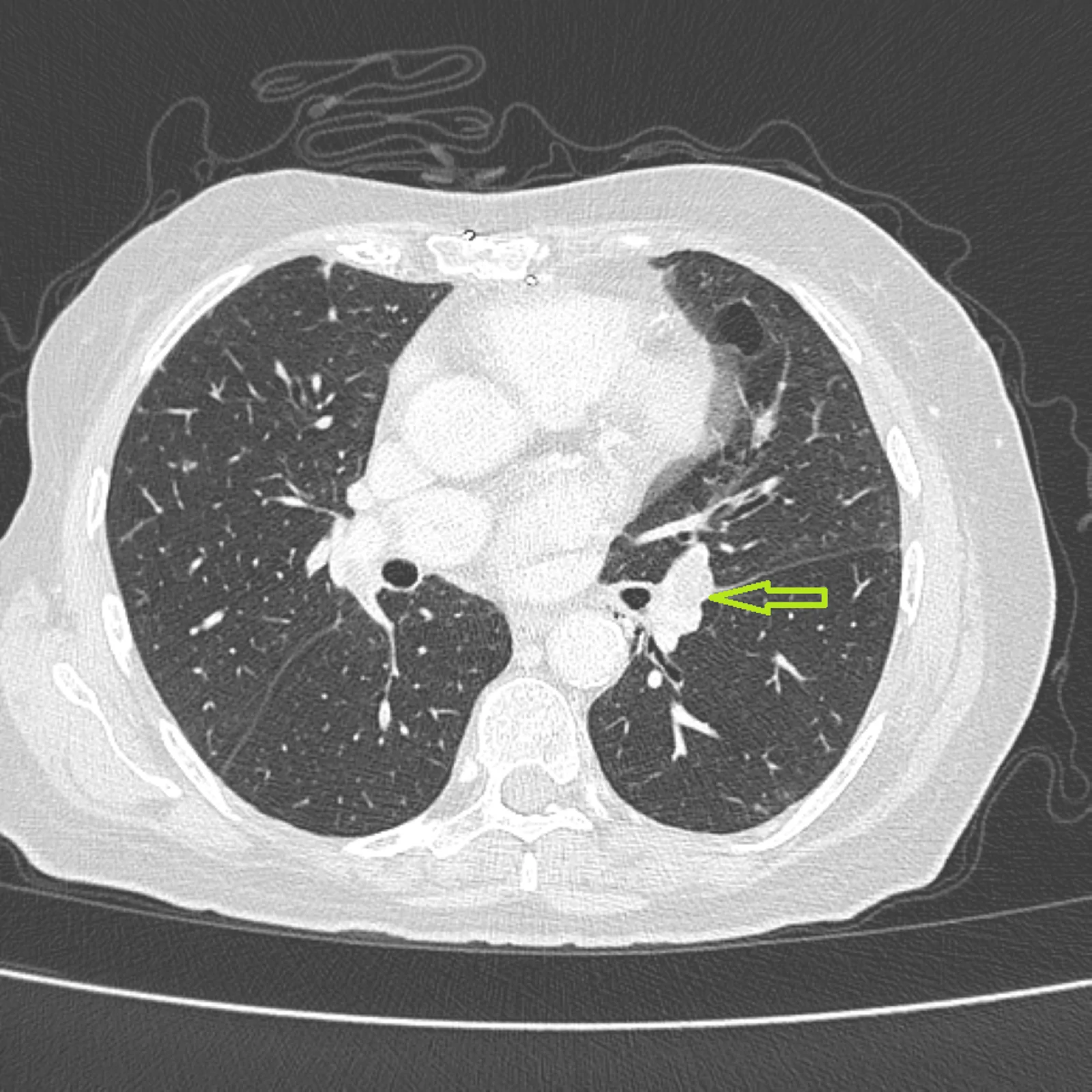
Cross-sectional image of CT thorax with contrast, the green arrow pointing towards the largest visible lymph node (reactive vs. metastatic deposit).

Figure 7 corresponds to a cross-sectional image of CT thorax showing enlarged lymph nodes that were deemed secondary to either multiple pulmonary embolisms or metastatic deposits.

**Figure 8:**
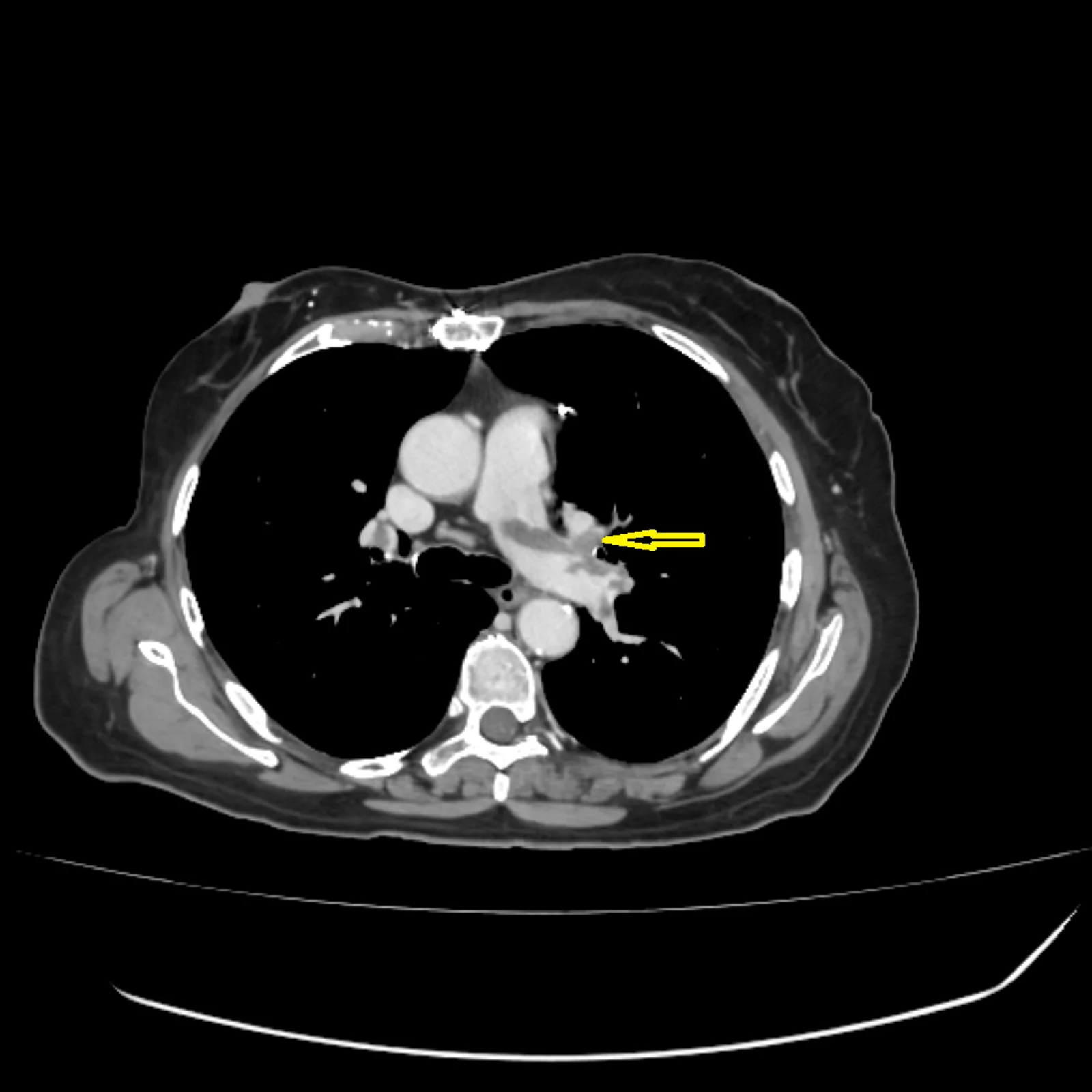
Cross-sectional image of CT thorax with contrast, the yellow arrow pointing towards saddle pulmonary embolism.

Figure 8 corresponds to a cross-sectional image of CT thorax with contrast showing saddle pulmonary embolism.

**Figure 9:**
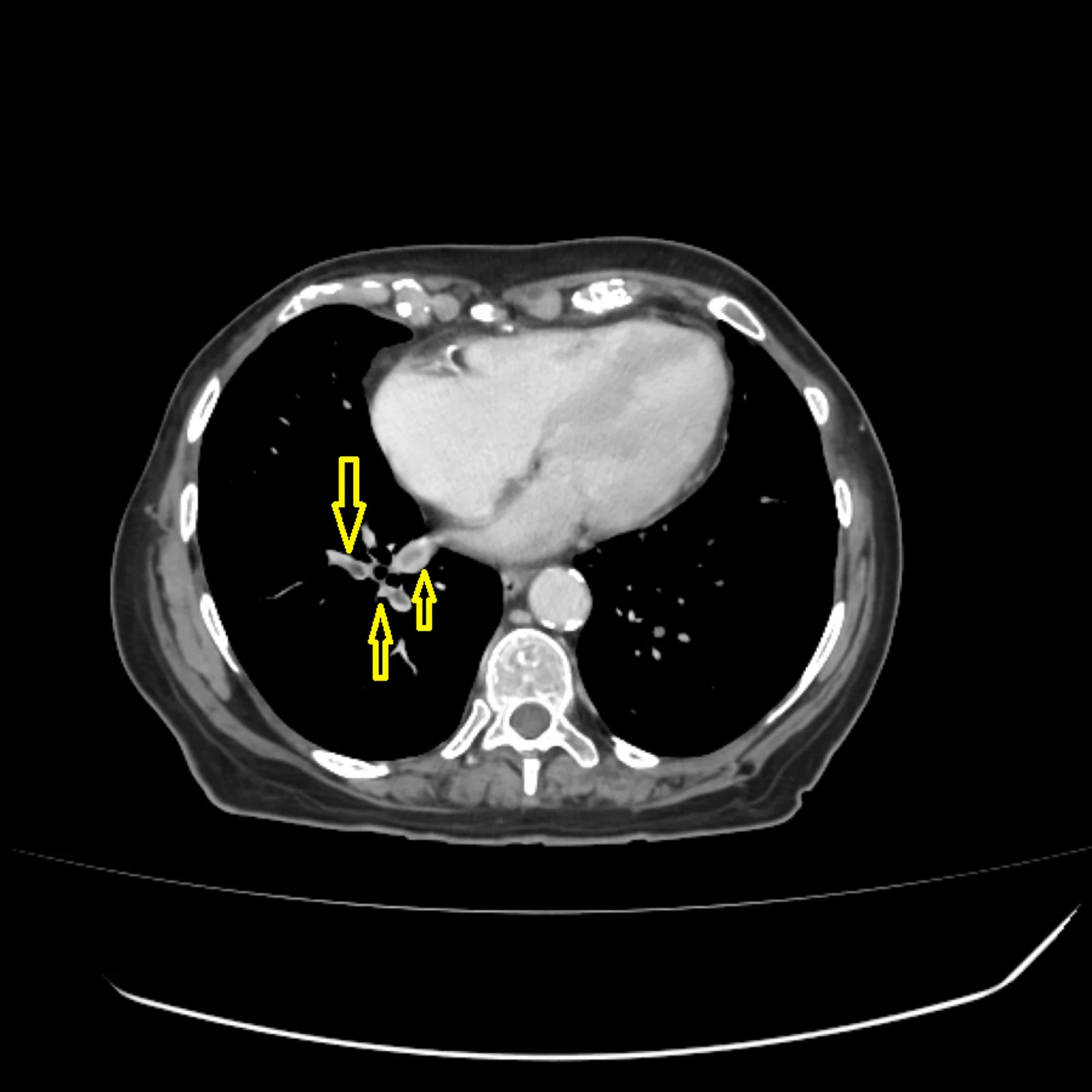
Cross-section of CT thorax with contrast, the yellow arrows pointing towards multiple small embolisms in pulmonary vasculature.

Figure 9 corresponds to a cross-sectional image of CT thorax with contrast showing multiple subsegmental pulmonary embolisms.

**Figure 10:**
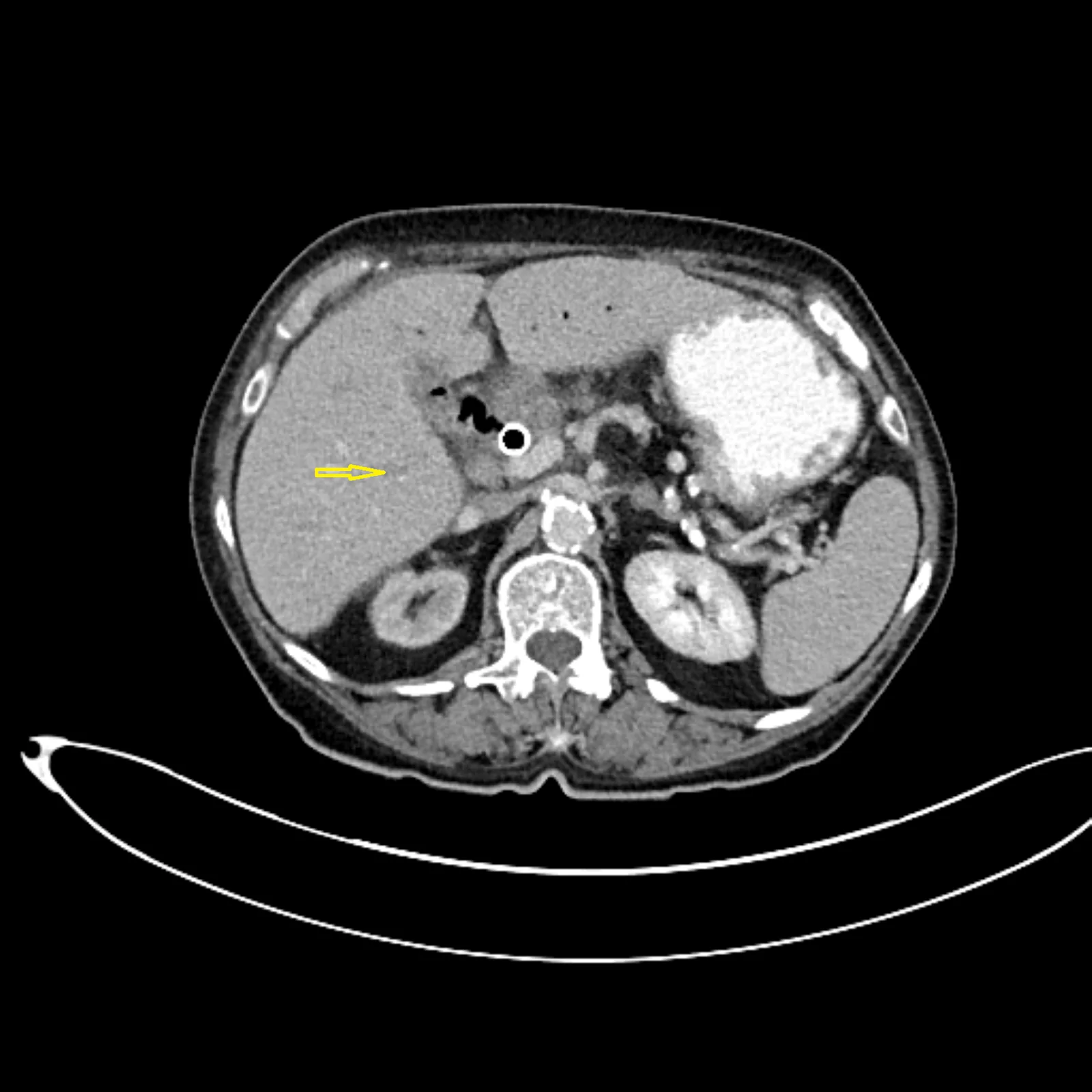
Cross-section of CT abdomen with contrast, the yellow arrow pointing towards multiple liver hypodensities consistent with metastatic disease.

Figure 10 corresponds to a cross-sectional image of CT abdomen with contrast showing hepatic metastasis.

The patient was commenced on therapeutic anticoagulation and referred to the hepato-pancreato-biliary multidisciplinary team and oncology services for further management.

## Discussion

Diabetes is considered to be a modifiable risk factor for pancreatic cancer [4]. Studies also show that pancreatic cancer is frequently associated with disturbances in glucose metabolism, including both new-onset diabetes and worsening glycaemic control [1-3]. The underlying mechanisms are thought to involve tumour-induced insulin resistance and beta-cell dysfunction [5]. It can be secondary to paraneoplastic syndrome. In some cases, deterioration in glycaemic control may precede the clinical detection of malignancy. Resection of pancreatic cancer has also been shown to improve glycaemic control in already known diabetic patients [1].

A key feature of this case is the presence of a normal contrast-enhanced CT scan performed four months prior to diagnosis. This highlights the limitations of imaging in detecting early pancreatic malignancy. Previous studies have demonstrated that pancreatic cancer may be radiologically occult in its early stages, with subtle abnormalities often missed on initial imaging [7,8].

The concept of interval pancreatic cancer, defined as malignancy diagnosed shortly after negative imaging, is increasingly recognised [7]. This case illustrates the aggressive nature of pancreatic cancer and its potential to progress rapidly from undetectable disease to metastatic cancer within a short period.

Additionally, the presence of a saddle pulmonary embolism is consistent with the prothrombotic state associated with malignancy, often referred to as Trousseau syndrome [9,10]. This further supports the systemic impact of pancreatic cancer.

Importantly, this case emphasises the role of clinical judgement. In elderly patients, worsening diabetes is often attributed to non-compliance or lifestyle factors. However, in this case, careful history-taking and recognition of an atypical presentation prompted further investigation, leading to the diagnosis of an underlying malignancy despite recent reassuring imaging.

## Conclusions

Rapid deterioration of previously well-controlled diabetes in patients with good compliance with anti-glycaemic medication should prompt consideration of underlying malignancy, particularly pancreatic cancer, especially in older adults, as diabetes is considered to be a modifiable risk factor for developing pancreatic cancer. Though this patient did not present with typical vague symptoms of weight loss for malignancy but history tells us that she had new-onset iron deficiency anaemia with normal radiology. This could have been an early sign of malignancy and helps us conclude that pancreatic cancer is not always visible on CT scan in very early stages. This also shows that normal recent radiology should not deter us from excluding the diagnosis of pancreatic cancer. Clinical suspicion should guide further investigation when the presentation is unexplained or atypical.
